# Supplementary material for: The Impact of PSMA-PET on Oncologic Control in Prostate Cancer Patients Who Experienced PSA Persistence or Recurrence
Source: Cancers (Basel). 2022 Dec 30;15(1):247. doi: 10.3390/cancers15010247 (PMC9818949; doi:10.3390/cancers15010247)
Supplement: Supplementary file 1 [file cancers-15-00247-s001.zip › cancers-2073123-supplementary.pdf]

Supplementary Materials: The impact of PSMA-PET on Oncologic Control in Prostate Cancer Patients Who Experienced PSA Persistence or Recurrence

Lorenzo Bianchi, Francesco Ceci, Francesco Costa, Eleonora Balestrazzi, Matteo Droghetti, Pietro Piazza, Alessandro Pissavini, Riccardo Mei, Andrea Farolfi, Paolo Castellucci, Stefano Puliatti, Alessandro Larcher, Giorgio Gandaglia, Daniele Robesti, Alexandre Mottrie, Alberto Briganti, Alessio Giuseppe Morganti, Stefano Fanti, Francesco Mortorsi, Riccardo Schiavina and Eugenio Brunocilla

Table S1. Descriptive characteristics in patients with positive PSMA-PET (*n* = 193), in the pre-salvage (*n* = 69) and post-salvage (*n* = 124) setting, stratifying according to localization of disease at PSMA-PET (namely, local and/or N1 vs. M1).

	**Pre-salvage setting, *n* = 69 (36%)**			**Post-salvage setting, *n* = 124 (64%)**		
	**Local and/or N1**	**M1**	***p* value**	**Local and/or N1**	**M1**	***p* value**
Patients, *n* (%)	31 (44.9)	38 (55.1)		66 (53.2)	58 (46.8)	-
Age<br>Median (IQR)	63 (59–70)	64 (57–69)	0.8	62 (56–66)	62 (58–68)	0.4
PSA level at RP (ng/ml)<br>Median (IQR)	6.9 (6–9.8)	8.25 (5.45–20.5)	0.7	9.7 (7–13.9)	10.7 (4.6–18)	0.8
pT stage, *n* (%)			0.001			0.03
pT2	10 (32.3)	2 (5.3)		25 (37.9)	15 (25.9)	
pT3a	13 (41.9)	10 (26.3)		25 (37.9)	16 (27.6)	
pT3b–pT4	8 (25.8)	26 (68.4)		16 (24.2)	27 (46.6)	
pN stage, *n* (%)			0.4			0.04
pNx	5 (16.1)	4 (10.5)		21 (31.8)	9 (15.5)	
pN0	17 (54.8)	17 (44.7)		34 (51.5)	30 (51.7)	
pN1	9 (29)	17 (44.7)		11 (16.7)	19 (32.8)	
Pathologic ISUP group, *n* (%)			0.002			≤ 0.001
ISUP 1-3	17 (54.8)	7 (8.4)		41 (62.1)	17 (29.3)	
ISUP 4-5	14 (45.2)	31 (81.6)		25 (37.9)	41 (70.7)	
Adjuvant Radiotherapy, *n* (%)			0.055			≤ 0.001
Yes	5 (16.1)	14 (36.8)		11 (16.7)	27 (46.6)	
No	26 (83.9)	24 (3.2)		55 (83.3)	31 (53.4)	
PSA level at PET PSMA, ng/ml<br>Median (IQR)	0.4 (0.21–1.55)	0.84 (0.4–2.3)	0.1	0.62 (.26–1.3)	0.69 (0.4–1.65)	0.3

PSMA: Prostate Specific Membrane Antigen; PET: Positron Emission Tomography; IQR: interquartile range; PSA: prostate specific antigen; RP: radical prostatectomy; ISUP: International Society of Urological Pathology.

Table S2. Descriptive characteristics in patients with positive PSMA-PET (n = 193), in the pre-salvage (n = 69) and post-salvage (n = 124) setting stratifying according to stage of disease at PSMA-PET (namely, oligorecurrent vs polirecurrent).

	**Pre-salvage setting, n = 69 (36%)**			**Post-salvage setting, n = 124 (64%)**		
	**Oligorecurrent (≤3 lesions)**	**Polirecurrent (>3 lesions)**	**p value**	**Oligorecurrent (≤3 lesions)**	**Polirecurrent (>3 lesions)**	**p value**
Patients, n (%)	62 (90)	7 (10)	-	113 (91)	11 (9)	-
Age<br>Median (IQR)	63 (59–68)	72 (68–74)	0.02	62 (56–66)	68 (61–73)	0.048
PSA level at radical treatment (ng/ml)<br>Median (IQR)	6.9 (6–16)	8 (6.8 -	0.07	9.38 (5.6–14.8)	13.45 (5.95–30)	0.2
pT stage, n (%)			0.02			0.1
pT2	12 (19.4)	0		38 (33.6)	2 (18.2)	
pT3a	23 (37.1)	0		39 (34.5)	2 (18.2)	
pT3b–pT4	27 (43.5)	7 (100)		36 (31.9)	7 (63.6)	
pN stage, n (%)			0.3			0.04
pNx	7 (11.3)	2 (28.6)		29 (25.7)	1 (9.1)	
pN0	32 (51.6)	2 (28.6)		69 (53.1)	4 (36.4)	
pN1	23 (37.1)	3 (42.9)		24 (21.2)	6 (54.5)	
Pathologic ISUP group, n (%)			0.04			0.2
ISUP 1-3	24 (38.7)	0		55 (48.7)	3 (27.3)	
ISUP 4-5	38 (61.3)	7 (100)		58 (51.3)	8 (72.7)	
Adjuvant Radiotherapy, n (%)			0.06			0.002
Yes	15 (24.2)	4 (57.1)		30 (26.5)	8 (72.7)	
No	47 (75.8)	3 (42.9)		83 (73.5)	3 (27.3)	
PSA level at PET PSMA, ng/ml<br>Median (IQR)	0.7 (0.3–1.66)	1.9 (0.87–3.16)	0.07	0.6 (0.3–1.3)	1.8 (0.5–3.7)	0.009

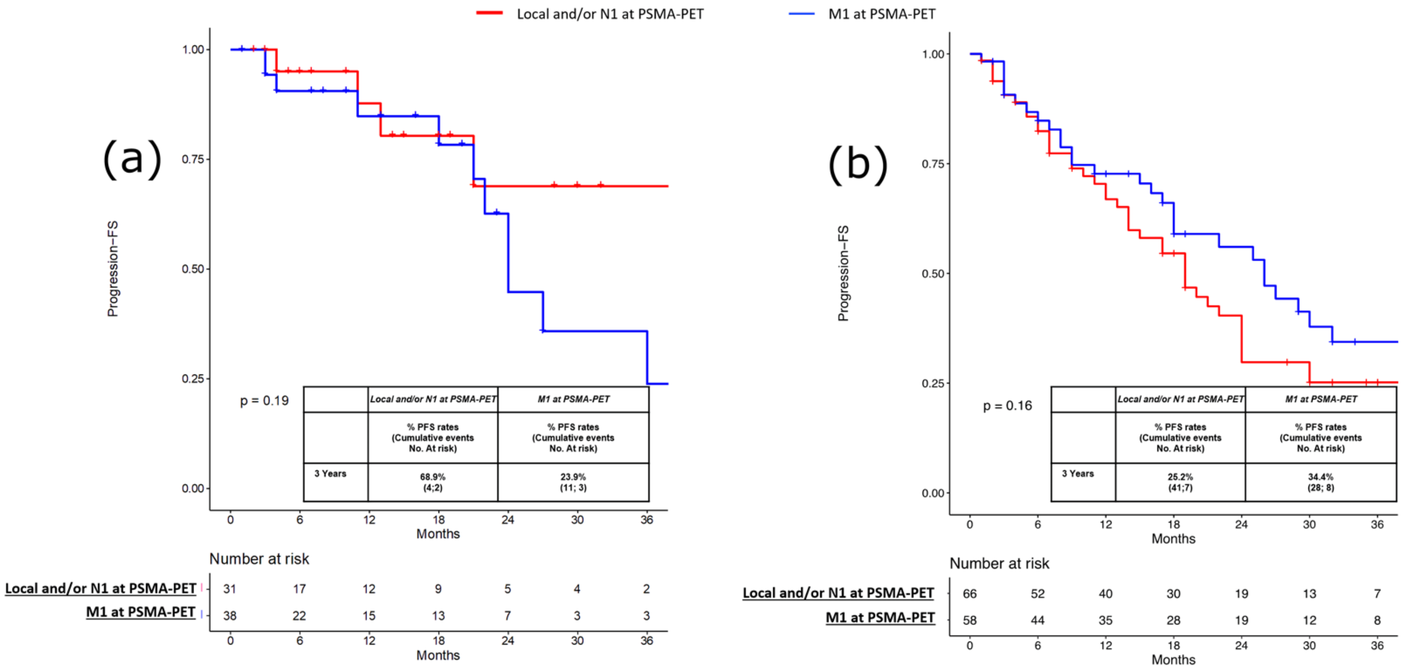

Figure S1. Kaplan-Meier curves depicting Progression Free Survival (PFS) rates of patients with positive PSMA-PET (a) in the pre-salvage setting ($n = 69$) and (b) in the post-salvage setting ($n = 124$) according to anatomic sites of recurrence depicted at PSMA-PET (namely, local and/or N1 vs. M1).

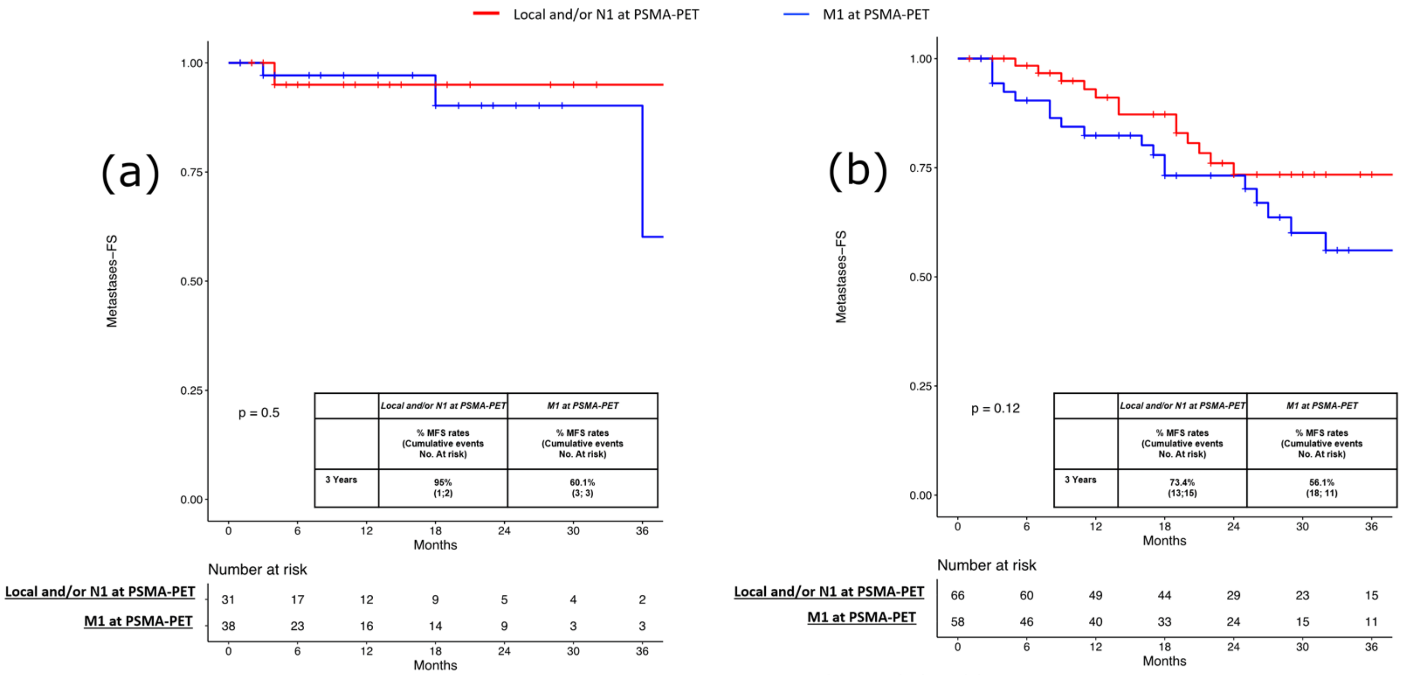

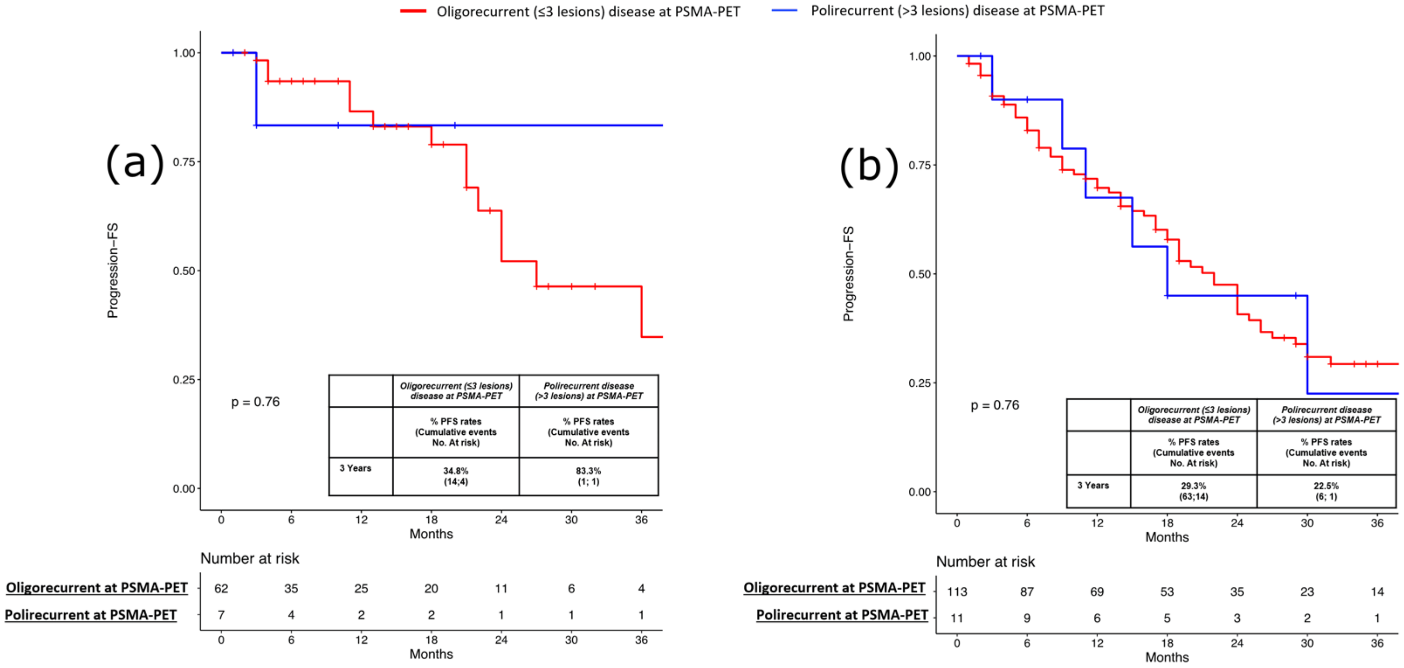

Figure S2. Kaplan-Meier curve depicting Metastases Free Survival (MFS) rates of patients with positive PSMA-PET (a) in the pre-salvage setting ($n = 69$) and (b) in the post-salvage setting ($n = 124$) according to anatomic sites of recurrence depicted at PSMA-PET (namely, local and/or N1 vs. M1).

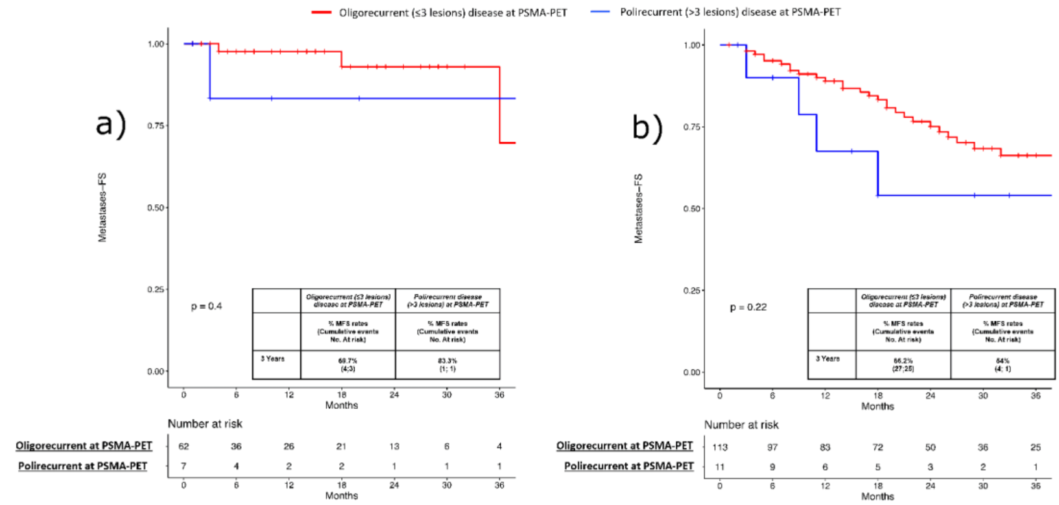

Figure S3. Kaplan-Meier curves depicting Progression Free Survival (PFS) rates of patients with positive PSMA-PET (a) in the pre-salvage setting (n = 69) and (b) in the post-salvage setting (n = 124) according to number of lesions depicted at PSMA-PET (namely, oligo-recurrent [≤ 3 lesions] vs. polirecurrent Pca [>3 lesions].

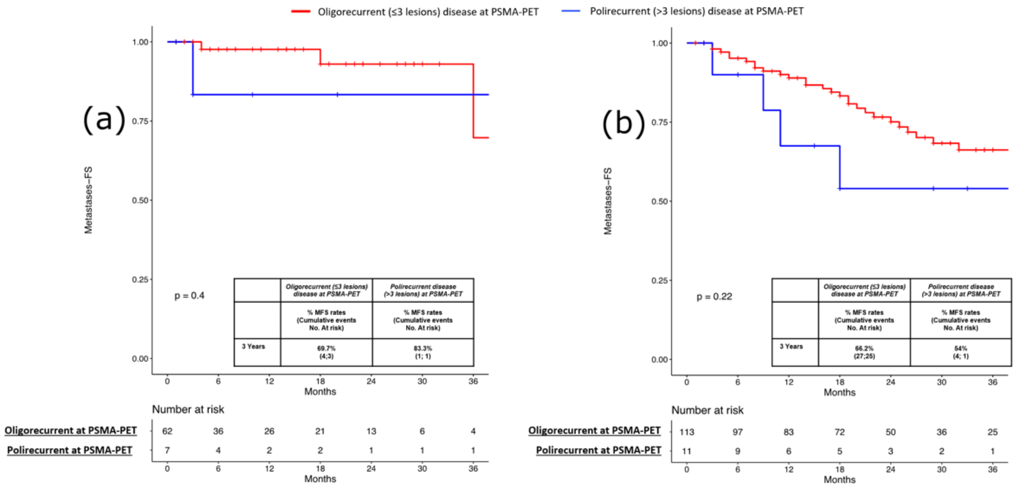

Figure S4. Kaplan-Meier curve depicting Metastases Free Survival (MFS) rates of patients with positive PSMA-PET (a) in the pre-salvage setting (n = 69) and (b) in the post-salvage setting (n = 124) according to number of lesions depicted at PSMA-PET (namely, oligo-recurrent [≤ 3 lesions] vs. polirecurrent Pca [>3 lesions].
